# Antibiotic Streptolydigin Requires Noncatalytic Mg^2+^ for Binding to RNA Polymerase

**DOI:** 10.1128/AAC.02248-13

**Published:** 2014-03

**Authors:** Savva Zorov, Yulia Yuzenkova, Vadim Nikiforov, Konstantin Severinov, Nikolay Zenkin

**Affiliations:** a Centre for Bacterial Cell Biology, Institute for Cell and Molecular Biosciences, Newcastle University, Newcastle upon Tyne, United Kingdom; b Waksman Institute, Rutgers, the State University of New Jersey, Piscataway, New Jersey, USA; c Faculty of Bioengineering and Bioinformatics, Moscow State University, Moscow, Russia; d Public Health Research Institute Center, New Jersey Medical School at UMDNJ, Newark, New Jersey, USA; e Skolkovo Institute of Science and Technology, Skolkovo, Russia; f St. Petersburg State Polytechnical University, St. Petersburg, Russia

## Abstract

Multisubunit RNA polymerase, an enzyme that accomplishes transcription in all living organisms, is a potent target for antibiotics. The antibiotic streptolydigin inhibits RNA polymerase by sequestering the active center in a catalytically inactive conformation. Here, we show that binding of streptolydigin to RNA polymerase strictly depends on a noncatalytic magnesium ion which is likely chelated by the aspartate of the bridge helix of the active center. Substitutions of this aspartate may explain different sensitivities of bacterial RNA polymerases to streptolydigin. These results provide the first evidence for the role of noncatalytic magnesium ions in the functioning of RNA polymerase and suggest new routes for the modification of existing and the design of new inhibitors of transcription.

## INTRODUCTION

DNA-dependent RNA polymerase (RNAP) is a potent target for antibiotics. At present, two specific inhibitors of bacterial RNAPs, rifampin and lipiarmycin (fidaxomicin), are in clinical use as antibiotics, and there is still great potential for other known inhibitors of bacterial RNAPs (or their derivatives) to be used in the clinic in the future.

**FIG 1:**
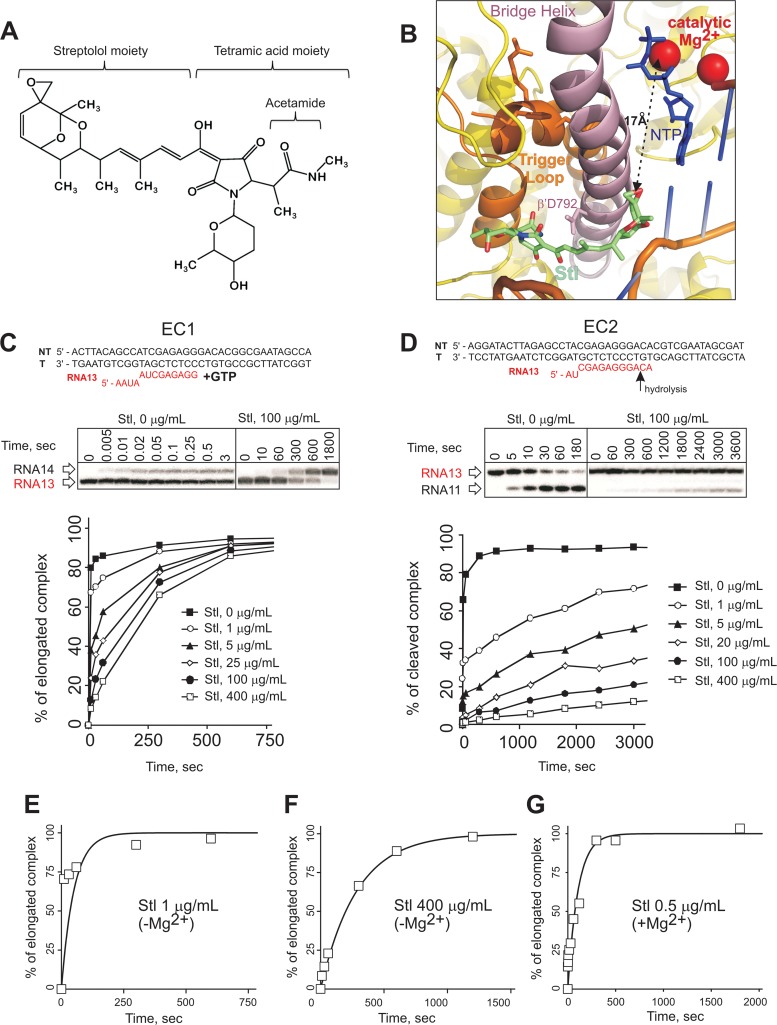
Inhibition of elongation and intrinsic cleavage of RNA by Stl. (A) Chemical structure of Stl. (B) Close-up view of Stl bound in the active center in the crystal structure of the T. thermophilus RNAP elongation complex (Protein Data Bank [PDB] code 2PPB). The β subunit was removed for clarity. The amino acids of the TL (orange), mutated in this study, are shown as orange sticks. (C and D) Schemes of the elongation complexes (EC1 and EC2) used and representative phosphorimaging scans of the products of the reactions separated in denaturing polyacrylamide gels are shown above the plots. T, template strands; NT, nontemplate strands. RNA (red) was radiolabeled at the 5′ end. (C) Kinetics of GTP incorporation (1 mM GTP and 10 mM Mg^2+^) in EC1 in the presence of different concentrations of Stl. (D) Kinetics of intrinsic (endonucleolytic) cleavage (10 mM MgCl_2_) in EC2 in the presence of different concentrations of Stl. Note that the addition of nonsaturating Stl before the reactants results in two fractions (fast and slow) of the elongation complexes. (E to G). Kinetics of NMP incorporation in the presence of different concentrations of Stl, preincubated with or without Mg^2+^, were fitted in a single-exponent equation. Note the clearly double exponential nature of the kinetics data in panel E.

The antibiotic streptolydigin (Stl) is a derivative of 3-acetyltetramic acid (Fig. 1A), and it has been known for a long time to specifically inhibit bacterial RNAPs (1–3). Stl does not inhibit eukaryotic RNAPs, although their structural similarity with bacterial RNAPs is high (4–6). Stl demonstrates only partial cross-resistance with the antibiotic rifampin, which is in wide clinical use (7), and some other known inhibitors of bacterial RNAPs, such as microcin J25 (8–10), CBR703 (11), and sorangicin (12). Besides being of interest for drug development, Stl as an inhibitor of the RNAP active center (below) is useful for a fundamental understanding of the catalytic mechanisms of transcription.

The crystal structures of Stl complexed with the Thermus thermophilus core RNAP (13, 14) and the T. thermophilus elongation complex (15) revealed that the antibiotic binds along the bridge helix (BH) about 20 Å away from the catalytic Mg^2+^ ions of the active center (Fig. 1B), which participate in catalysis of all the reactions performed by the RNAPs (16, 17). Structural and biochemical analyses showed that Stl freezes the unfolded conformation of a flexible domain of the active center, the trigger loop (TL) (Fig. 1B). The TL was later shown to be essential for catalysis of all reactions by the active center (18–20), explaining the ability of Stl to inhibit all RNAP catalytic activities (13).

The two largest subunits, β and β′, are involved in the binding of Stl (13, 21–24). The binding site is formed on the “DNA side” of the bridge helix (Fig. 1B); the streptolol moiety of Stl interacts with regions STL1 (positions 538 to 552 of the second-largest subunit; β^538–552^ [Escherichia coli numbering]) and STL2 (β^557–576^) and the N-terminal portion of the BH (β′^769–788^) (13), while the tetramic acid groups interact with the central portion of the BH (β′^789–795^) and with the ordered segment of the TL (13). The acetamide group of the tetramic acid moiety of Stl and β′D792 of the BH are critical for Stl binding (13, 24). Here we provide evidence that the binding of Stl to RNAP strictly requires a noncatalytic Mg^2+^ ion, which apparently bridges the Stl tetramic acid moiety to β′D792 of the BH. To the best of our knowledge, this is the first direct evidence for the role of noncatalytic Mg^2+^ ions in RNAP functioning.

## MATERIALS AND METHODS

### WT and mutant RNAPs.

Recombinant wild-type (WT) and mutant Thermus aquaticus core RNAPs were constructed and purified as described previously (25).

### Transcription essays.

Elongation complexes (ECs) were assembled with WT and mutant (H936A/R933A and M932A [E. coli numbering]) RNAPs as described previously (18) and placed in transcription buffer containing 40 mM KCl and 20 mM Tris (pH 7.9). Prior to complex assembly, RNA was ^32^P labeled at the 5′ end by using [γ-^32^P]ATP (PerkinElmer). All reactions were carried out at 40°C. Stl (Sigma) with or without 10 mM MgCl_2_ was added before the reactions for 10 min at 40°C. Elongation reactions were initiated by addition of 1 mM GTP or 1 mM GTP with 10 mM MgCl_2_; endonucleolytic cleavage reactions were initiated by the addition of 10 mM MgCl_2_. Reactions were stopped by the addition of formamide-containing buffer, and products were analyzed as described previously (18). Fast kinetics experiments were performed as described previously (18). The kinetics data that were described well by a single exponent were fitted into a single-exponent equation using the nonlinear regression procedure in SigmaPlot software. Plots were normalized to the predicted maximum, which was taken as 100. The rates were then fitted in a hyperbolic equation to determine the *K_i_*[Stl]. When the kinetics was described by a double exponent, the predicted maxima of the fast fractions were used for the fit to determine the Stl concentration that inhibited half of the complexes (equivalent of the *K_i_*).

### Mg^2+^ binding to Stl.

The spectra of Stl were recorded using a UV160U UV-visible (UV-Vis) recording spectrophotometer (Shimadzu Scientific Instruments, Inc.) in a fused silica measuring cell in the presence of the indicated concentrations of the MgCl_2_ in 20 mM HEPES buffer (pH 8.0).

## RESULTS

In order to investigate the details of the effects of Stl on RNAP active center functioning, we used artificially assembled elongation complexes (ECs). These complexes are assembled with Thermus aquaticus RNAP, fully complementary synthetic template and nontemplate DNA oligonucleotides, and an RNA oligonucleotide (Fig. 1C and D). The complexes are indistinguishable from “native” elongation complexes obtained by transcription from a promoter (19, 26, 27). We analyzed the kinetics of nucleoside monophosphate (NMP) addition in the presence of different concentrations of Stl in the elongation complex EC1, containing a 13-nucleotide-long RNA (Fig. 1C). Note that Stl was preincubated with the EC for 10 min before the addition of nucleoside triphosphate (NTP) with Mg^2+^, which initiated synthesis. Curiously, at lower concentrations of Stl, we observed a clear separation of the kinetics curve into two phases, fast and slow (Fig. 1C and E, the latter as an example of the fit of the data into a single-exponent equation). At higher concentrations, the division was less obvious, and the kinetics can be described by a single-exponent equation (Fig. 1F).

Next, we tested the effects of increasing concentrations of Stl on the hydrolysis of the phosphodiester bond, a reaction also catalyzed by the RNAP active center. As seen from Fig. 1D, Stl also divided the ECs into sensitive (slow) and resistant (fast) fractions. Interestingly, however, the concentration of Stl required to inhibit half of the ECs (the inhibition constant *K_i_* cannot be used in this case, given that the kinetics cannot be described by a single-exponent equation) during hydrolysis was ∼8 times lower than that during NTP addition (Table 1).

**TABLE 1:** Correlation of the rate of the reaction and efficiency of inhibition by Stl preincubated with or without Mg^2+^

RNAP	Reaction (*k*_obs_) (s^−1^)	*K_i_*[Stl] (μg/ml)
WT		
1 mM GTP incorporation		
−Mg^2+^	100 ± 19	8.32 ± 1.08^*a*^
+Mg^2+^		0.53 ± 0.036
Hydrolysis, −Mg^2+^	0.049 ± 0.007	1.1 ± 0.15^*a*^
H936A/R933A mutant		
1 mM GTP incorporation, −Mg^2+^	0.037 ± 0.004	0.49 ± 0.14
M932A mutant		
1 mM GTP incorporation, −Mg^2+^	0.028 ± 0.003	0.39 ± 0.06

a Half-inhibitory concentration determined from the proportion of the fast fraction.

The rate of phosphodiester bond hydrolysis is much lower than that of NMP incorporation (Table 1). We hypothesized that, upon the start of the reaction by addition of reactants, Stl requires time to manifest its inhibitory activity (note that complexes were preincubated with Stl before the start of the reaction), which would explain the more successful inhibition of the slower reaction of hydrolysis by Stl. To test this hypothesis, we analyzed the inhibition of RNA extension by mutant RNAPs (β′H936A/R933A and β′M932A) with strongly decreased rates of phosphodiester bond formation (18). β′H936/R933 of the TL participates in the catalysis of the phosphodiester bond formation by stabilizing the transition state of the reaction (18). β′M932 of the TL stacks on the base of the NTP bound in the active center and stabilizes the TL in the folded state, which is required for catalysis (18). Accordingly, the alanine substitutions of either β′H936/R933 or β′M932 drastically slow down NMP incorporation, although through different mechanisms (18). Neither of these amino acids participates in the Stl binding (Fig. 1B) (13, 18). We argued that, upon addition of Mg^2+^ and NTP, Stl would have enough time to inhibit ECs before the reaction had started. Indeed, as shown in Table 1, the half-inhibitory concentrations of Stl for NMP incorporation by β′H936A/R933A and β′M932A RNAPs were 15 to 20 times lower than that for WT RNAP. Given that the mutations slow down the reaction via different ways, the result suggests that the slowness of the mutant RNAPs leads to their Stl sensitivity. Note also a correlation between the inhibitory effects of Stl and the rates of the reactions in Table 1.

The above results suggest that the inhibition by Stl begins to establish itself only after the addition of the reactants. The only common component added to start the NMP incorporation and hydrolysis is Mg^2+^. We hypothesized that Mg^2+^ is required for Stl binding to RNAP. According to this proposal, when Mg^2+^ is added together with NTP, Stl does not have time to bind to all WT ECs before the reaction in them has already finished, thus giving birth to the fast and slow fractions of ECs. The hypothesis predicts that the preincubation of ECs with Mg^2+^ and Stl before the addition of NTPs would result in more efficient inhibition. To test this prediction, we preincubated WT ECs with Stl and Mg^2+^ prior to the addition of NTP (a similar experiment cannot be duplicated for phosphodiester bond hydrolysis because the addition of Mg^2+^ would initiate the reaction). Indeed, as seen from Fig. 1G, the kinetics in the presence of even small concentrations of Stl can be described by a single-exponent equation. Accordingly, when Stl was preincubated with Mg^2+^, the half-inhibitory concentration of Stl was ∼15 times lower than that when Mg^2+^ was added together with NTP (Table 1). We therefore conclude that Stl binding to RNAP requires the Mg^2+^ ion.

**FIG 2:**
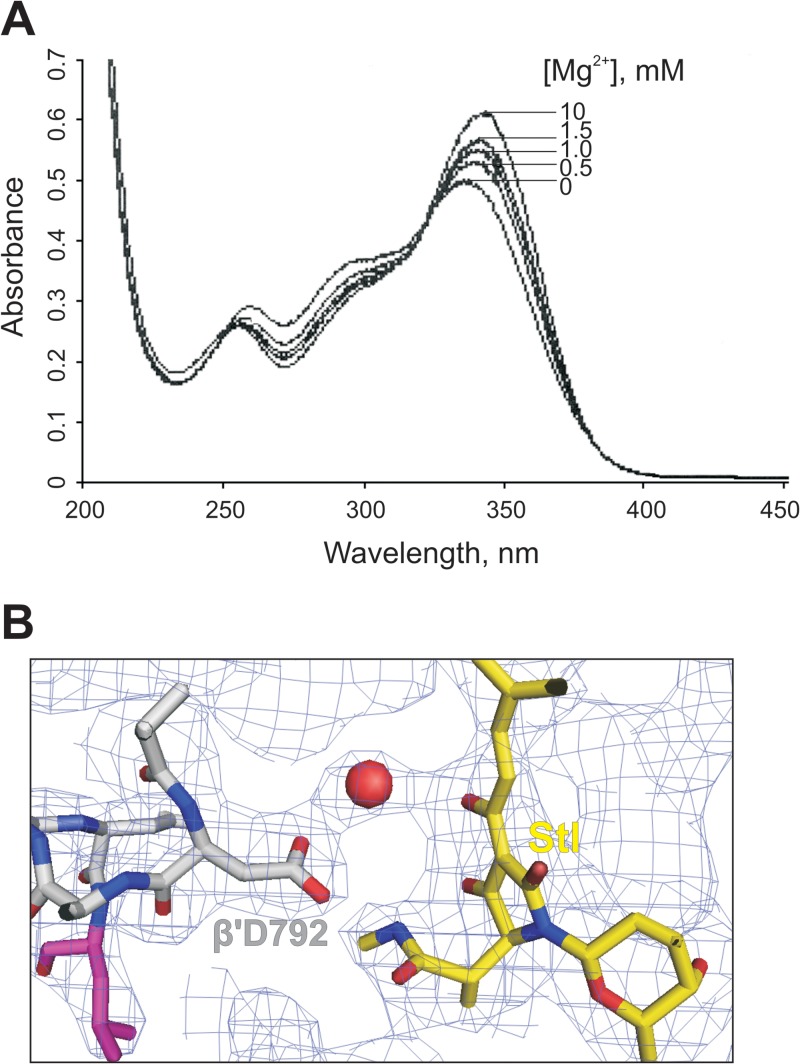
Inhibition of RNAP by Stl requires Mg^2+^. (A). Titration of Mg^2+^ onto Stl analyzed by UV absorption of Stl. (B) Unassigned electron density between the β′D792 (gray) and hydroxyl of the tetramic acid moiety of Stl (yellow) in the crystal structure of the T. thermophilus RNAP elongation complex (PDB code 2PPB), which can be attributed to Mg^2+^ (red circle).

We attempted to directly visualize if Stl can bind Mg^2+^. Stl has a distinct UV absorption spectrum. As seen from Fig. 2A, the spectrum undergoes marked changes upon titration with Mg^2+^. Together with the above results, this suggests that Stl may indeed chelate Mg^2+^, although whether the observed change in the Stl spectrum indeed accounts for Mg^2+^ binding requires further investigation.

## DISCUSSION

The principal finding of this study is that Stl requires Mg^2+^ to establish its inhibitory effect on RNAP. To the best of our knowledge, this is the first observation of a small-molecular-weight inhibitor of RNAP requiring an additional agent for its action. For example, the inhibitor tagetitoxin also binds in the vicinity of the active center and is known to chelate Mg^2+^ but does not require addition of external Mg^2+^, while the kinetics of NMP addition in the presence of tagetitoxin does not exhibit behavior similar to that observed with Stl (Fig. 1C and E) (28). The apparent affinity of Stl for Mg^2+^, as can be deduced from our titration experiment (Fig. 2A), is around 1 mM, which is close to the Mg^2+^ concentration in the bacterial cell. It is likely, however, that RNAP also provides binding determinants for the Mg^2+^ ion. Stl binds too far from the catalytic Mg^2+^ ions of the active site and is unlikely be influenced by them (Fig. 1B). However, careful examination of the crystal structure of the elongation complex bound to Stl reveals an electron density that is unaccounted for between the hydroxyl of the tetramic acid moiety of Stl and β′D792 of the BH, which can be attributed to Mg^2+^ (15) (Fig. 2B). This observation is consistent with the fact that the Stl analog tirandamycin, which lacks the acetamide group, is a significantly weaker inhibitor than Stl (29). Also, there are some other observations that point to the importance of the β′792 residue in Stl binding. Mutation β′D792G confers strong resistance to Stl (24). Furthermore, RNAPs that have aspartic acid at position β′792 (including *T. thermophilus and T. aquaticus* RNAPs) are much more sensitive to Stl than the RNAPs that have asparagine at this position (including E. coli RNAP). Finally, the β′N792D substitution in E. coli RNAP leads to a significant enhancement of the enzyme sensitivity to Stl (13). Taken together with our results, these facts suggest that Mg^2+^ bridges β′N792D and Stl, leading to tighter binding of the latter. This scenario also raises an intriguing possibility of a role for noncatalytic Mg^2+^ ions in the functioning/inhibition of the enzyme.
